# Analysis of contact pressure in a 3D model of dual-mobility hip joint prosthesis under a gait cycle

**DOI:** 10.1038/s41598-023-30725-6

**Published:** 2023-03-02

**Authors:** Mohammad Tauviqirrahman, Muhammad Imam Ammarullah, J. Jamari, Eko Saputra, Tri Indah Winarni, Febri Dwi Kurniawan, Shidnan Amir Shiddiq, Emile van der Heide

**Affiliations:** 1 Department of Mechanical Engineering, Faculty of Engineering, Diponegoro University, Semarang, 50275 Central Java Indonesia; 2 Department of Mechanical Engineering, Faculty of Engineering, Pasundan University, Bandung, 40153 West Java Indonesia; 3 Biomechanics and Biomedics Engineering Research Centre, Pasundan University, Bandung, 40153 West Java Indonesia; 4 Undip Biomechanics Engineering and Research Centre (UBM-ERC), Diponegoro University, Semarang, 50275 Central Java Indonesia; 5 Department of Mechanical Engineering, Semarang State Polytechnic, Semarang, 50275 Central Java Indonesia; 6 Department of Anatomy, Faculty of Medicine, Diponegoro University, Semarang, 50275 Central Java Indonesia; 7 Center for Biomedical Research (CEBIOR), Faculty of Medicine, Diponegoro University, Semarang, 50275 Central Java Indonesia; 8 Department of Mechanics of Solids, Surfaces and Systems (MS3), Faculty of Engineering Technology, University of Twente, Postbox 217, 7500 AE Enschede, The Netherlands; 9 Laboratory for Surface Technology and Tribology, Faculty of Engineering Technology, University of Twente, Postbox 217, 7500 AE Enschede, The Netherlands

**Keywords:** Biomaterials, Engineering, Materials science, Mathematics and computing

## Abstract

Hip joint prostheses are used to replace hip joint function in the human body. The latest dual-mobility hip joint prosthesis has an additional component of an outer liner that acts as a cover for the liner component. Research on the contact pressure generated on the latest model of a dual-mobility hip joint prosthesis under a gait cycle has never been done before. The model is made of ultrahigh molecular weight polyethylene (UHMWPE) on the inner liner and 316L stainless steel (SS 316L) on the outer liner and acetabular cup. Simulation modeling using the finite element method is considered static loading with an implicit solver for studying the geometric parameter design of dual-mobility hip joint prostheses. In this study, simulation modeling was carried out by applying varying inclination angles of 30°, 40°, 45°, 50°, 60°, and 70° to the acetabular cup component. Three-dimensional loads were placed on femoral head reference points with variations of femoral head diameter used at 22 mm, 28 mm, and 32 mm. The results in the inner surface of the inner liner, the outer surface of the outer liner, and the inner surface of the acetabular cup showed that the variations in inclination angle do not have a major effect on the maximum contact pressure value on the liner component, where the acetabular cup with an inclination angle of 45° can reduce contact pressure more than the other studied inclination angle variations. In addition, it was found that the 22 mm diameter of the femoral head increases the contact pressure. The use of a larger diameter femoral head with an acetabular cup configuration at a 45° inclination can minimize the risk of implant failure due to wear.

## Introduction

A dual-mobility hip prosthesis was introduced to reduce the risk of dislocation up to a 0.9% dislocation rate for 10 years of use^1^ and increase the overall stability and range of motion^2,3^. It was made to increase the range of motion when used on a daily basis^4^. An extended range of motion can avoid impingement in the hip joint prosthesis. Two general interactions that occur in a conventional dual-mobility hip joint prosthesis model are the acetabular cup with the liner and the liner with the femoral head, causing wear at two different locations^5,6^.

Ultrahigh molecular weight polyethylene (UHMWPE) is a widely used material, especially as a bearing material for hip joint replacement surgery^7,8^. The type of UHMWPE used in the medical field has a molecular weight ranging from 3.5 to 6 million g/mol and has a degree of crystallinity ranging from 50 to 55%^9^. In addition, metals are widely used in the orthopedic field, both in temporary and permanent equipment. The use of metal in permanent orthopedic equipment (prosthesis) cannot be separated from the consideration of chemical reactions that can occur when metal debris interacts with body tissues, especially bones^10–12^.

Adam et al.^13^ found that there are two kinds of wear. Wear as a result of convex surface interaction has a greater value than the one that occurs on a concave surface. Osteolysis and metallosis are caused by debris resulting from wear that occurs on dual-mobility hip joint prosthesis components^14,15^. To reduce the amount of polyethylene wear debris, a cover component made of metal was added to the liner so that the polyethylene convex surface did not experience major wear. Furthermore, the dual-mobility hip joint prosthesis model by Saputra et al.^16^ has an additional outer liner component that covers the entire polyethylene liner.

Experimental and clinical testing in assessing wear on dual-mobility hip joint prostheses requires higher costs, sophisticated equipment, and longer timeframes^17–19^. To avoid the obstacles faced by experimental and clinical studies, computational simulations based on the finite element method can be a strategic option^20,21^. This approach can also be the basis for initial research on the development of a dual-mobility hip joint prosthesis with various parameters so that it can assess the costs and energy wasted on trial-and-error efforts before continuing with experimental and clinical testing in further research. Computational simulation plays a key role in predicting contact pressure and efforts to reduce it with various parameters studied in a dual-mobility hip joint prosthesis. Contact pressure is an important aspect because it has a linear relationship with wear, which is one of the causes of implant failure^22,23^.

Walking is one of the most common daily human activities. The hip joint serves as the support and center of human walking movement^24^. The process of human walking is a cyclical pattern of limb movements that will determine the position of the human body^25^. The gait cycle is the period between two identical events in the gait process, which is used as a reference for the gait parameter test. During gait, humans generate different hip joint forces. The gait cycle and force terms are also widely used in several hip joint prosthesis-related studies^26,27^.

Based on the current model of dual-mobility hip prostheses^16^, contact pressure investigation by considering the gait cycle has not yet been carried out. The amount of information regarding this condition of contact pressure is necessary since walking gait is an everyday phenomenon. Therefore, the main purpose of the present study is to investigate the contact pressure of dual-mobility hip joint prostheses during the gait cycle.

## Materials and methods

### Material properties

The new dual-mobility prosthesis model by Saputra et al.^16^ consists of a femoral head, inner liner, outer liner, and acetabular cup. The femoral head was defined as an analytical rigid body. The inner liner was made of UHMWPE with Young’s modulus of 1 GPa and Poisson’s ratio of 0.4^28^. The outer liner and acetabular cup were made of 316L stainless steel (SS 316L) with Young’s modulus of 193 GPa and Poisson’s ratio of 0.3^29^.

### Geometry of dual-mobility hip joint prosthesis

**Figure 1:**
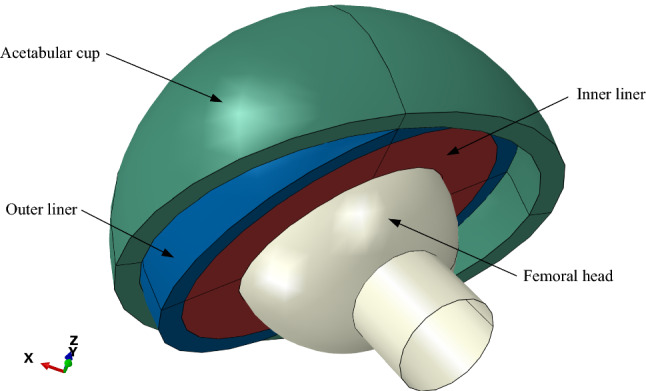
Geometry model of the present dual-mobility hip joint prosthesis.

**Figure 2:**
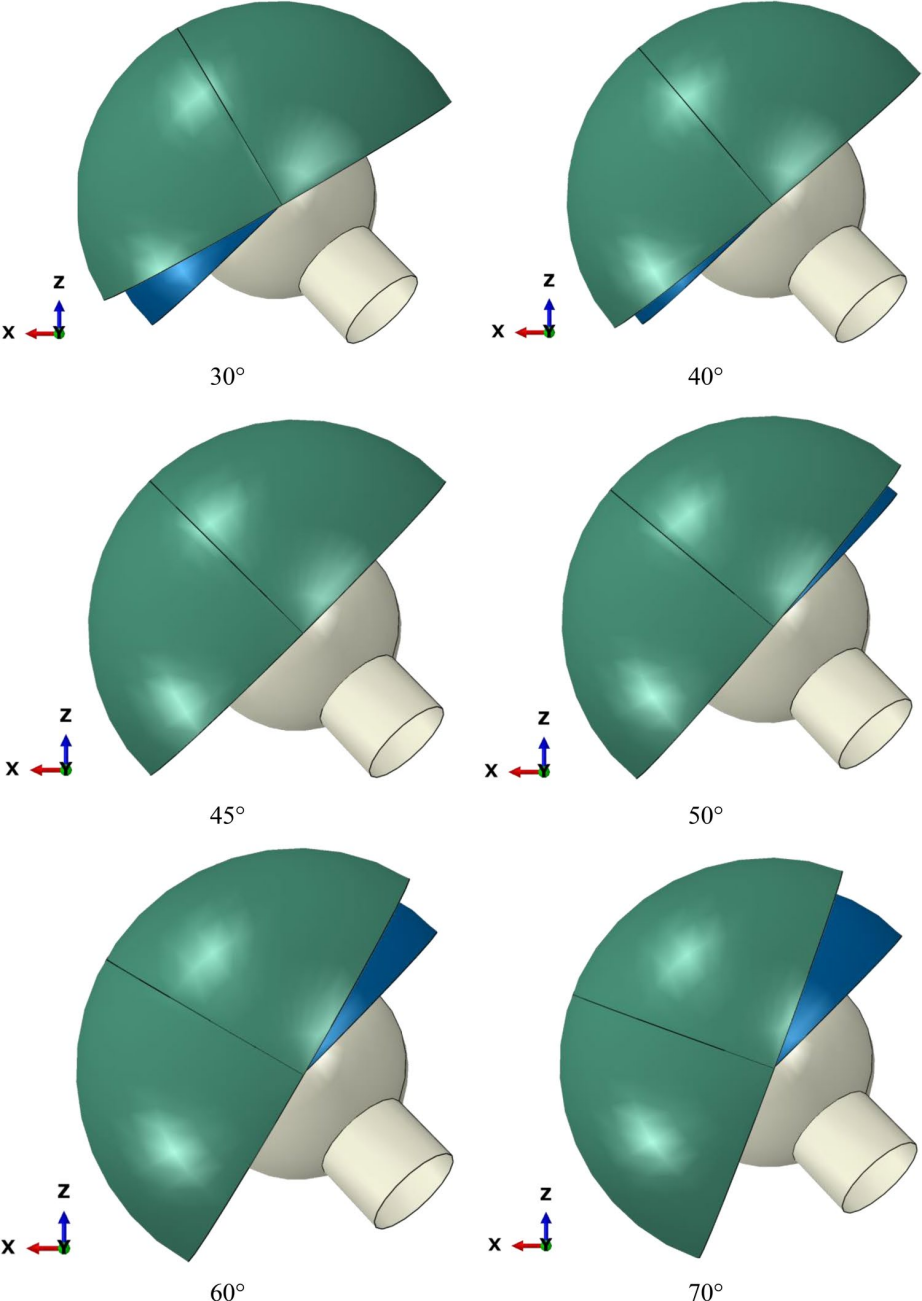
Variations in inclination angle on the acetabular cup component of the new dual-mobility hip joint prosthesis.

New geometric modeling was conducted based on the research by Saputra et al.^16^. The geometry of the dual-mobility hip joint prosthesis design was studied using ABAQUS CAE 2020 software considering static loading with an implicit solver. The geometries of the components are listed in Table 1 and were obtained by adopting the geometry of a commonly used single-mobility hip joint prosthesis with femoral head diameters of 22 mm, 28 mm, and 32 mm^30^ and then adopted for the design of the present dual-mobility hip joint prosthesis. The femoral head was defined to have an initial position based on the research conducted by Gao et al.^31^. The initial position was used to match the starting point of the walking gait cycle. The new dual-mobility hip joint prosthesis design is shown in Fig. 1. Variations in inclination angle were applied to the acetabular cup component as presented in Fig. 2. Based on the literature^32^, the inclination angle applied on the acetabular cup component would lead to different resulting contact pressure values on the bearing of the hip joint prosthesis. The inner liner and outer liner have the same ideal inclination on all variations of the acetabular cup inclination angle applied. There were six variations of inclination angle considered in the current dual-mobility hip joint prosthesis model that can occur in real conditions adopted from Gao et al.^31^ presented in Table 2.

**Table 1:** Geometry size of the dual-mobility hip joint prosthesis model.

Diameter (mm)							
Femoral head		Inner liner		Outer liner		acetabular cup	
Inner	Outer	Inner	Outer	Inner	Outer	Inner	Outer
–	22	22.2	34.2	34.2	39	39.2	44
–	28	28.2	40.2	40.2	45	45.2	50
–	32	32.2	44.2	44.2	49	49.2	54

**Table 2:** Inclination angles of the implant components.

Component	Angle
Liner	45°
Outer liner	45°
Cup	30°, 40°, 45°, 50°, 60°, and 70°

### Finite element model

**Figure 3:**
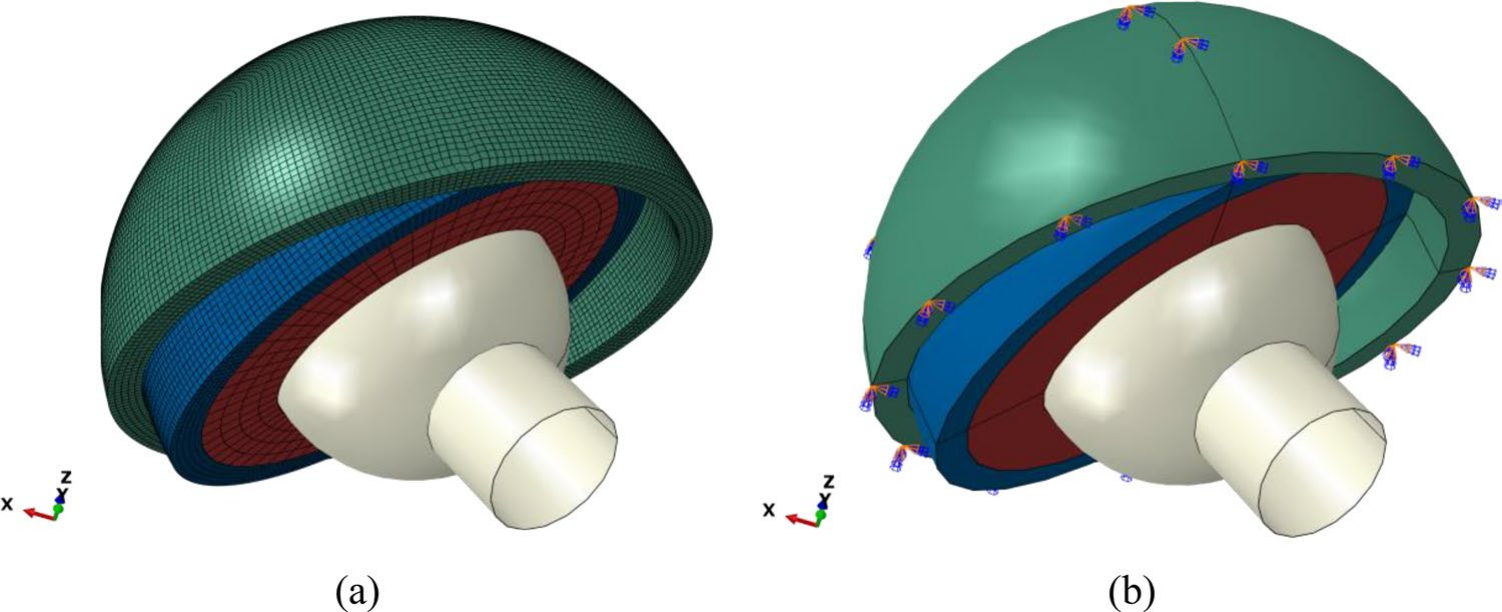
(**a**) Mesh of the model and (**b**) boundary condition for the case of a dual-mobility hip joint prosthesis.

In this work, the finite element method was used to predict the contact pressure of the dual-mobility hip joint prosthesis model. The 8-node hexahedron element type with structured hex mesh control was applied to all the components, as depicted in Fig. 3a. The inner liner, outer liner, and acetabular cup were meshed with element sizes of 1.5 mm, 0.5 mm, and 0.5 mm, respectively (the liner was approximately 3800 elements, the outer liner was approximately 68,000 elements, and the cup was approximately 85,000 elements). The number of elements has been considered in a computational model based on a convergence study using the H-refinement method^33,34^, where it is performed by generating smaller elements from initial meshing until a sufficient number of elements is obtained for an accurate result.

### Boundary condition of dual-mobility hip joint prosthesis

The boundary condition of the present dual-mobility hip joint prosthesis is reflected in Fig. 3b. The resultant forces from the gait cycle were applied at the reference point of the femoral head. Steady-state contact was performed in the current study between the acetabular cup, outer liner, inner liner, and femoral head, ignoring the possibility of micro separation and edge loading. The outer surface of the acetabular cup was fixed in all directions. The femoral head was considered to be able to move in a vertical direction without motion. Temperature changes during contact are not considered. The surface roughness in the contact interface from the inner surface of the acetabular cup with the outer surface of the outer liner and the inner surface of the inner liner with the outer surface of the femoral head is set to be frictionless. Then, the influence of the synovial fluid is absent in the context of a dry contact.

### Gait cycle

The three-dimensional concentrated quasistatic force at specific timepoints of the gait cycle was adopted based on research by Paul^35^. To simplify the computational simulation, the present computational simulation takes six specific timepoints of the gait cycle that represent the complete gait cycle, referring to the previous research by Ammarullah et al.^33,36^. The six specific timepoints describe several conditions: 0% is the beginning of the gait cycle, 20% is the second highest peak of the gait cycle, 35% is the lowest force before the end of the gait cycle, 50% is the middle of the gait cycle, 65% is the first highest peak, and 100% is the end of the gait cycle.

### Validation procedure

The computational model established in the present study needs to be verified with the previous literature to ensure the correctness of the results obtained. For this purpose, the computational simulation results will be compared with previous studies conducted by Gao et al.^31,37^ with single- and dual-mobility hip joint prosthesis models. The contact pressure values will be compared under identical conditions and parameters in terms of loading, material properties, and boundary conditions. If the comparison has a similar trend with relatively similar results (below 15% for every specific timepoint of the gait cycle), then the current model can be said to be valid with the successful verification of the results so that data analysis can be carried out.

## Results

### Validation

**Figure 4:**
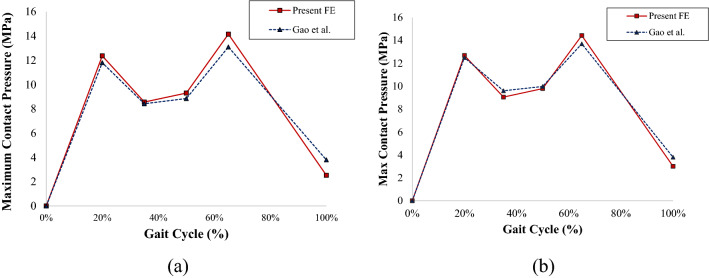
Contact pressure validation with Gao et al.^31,37^: (**a**) single-mobility, (**b**) dual-mobility hip joint prosthesis models.

Figure 4a,b show the comparison of contact pressure results between the present model and the published work of Gao et al.^31,37^. Based on Fig. 4, the results in a gait cycle between the current study and Gao et al.^31,37^ show a similar trend. The difference in contact pressure shows good agreement with the deviation of 6.38–11% for every specific timepoint of the gait cycle. The relatively small difference in results below 15% makes the current model valid.

### Contact pressure on the current model

**Figure 5:**
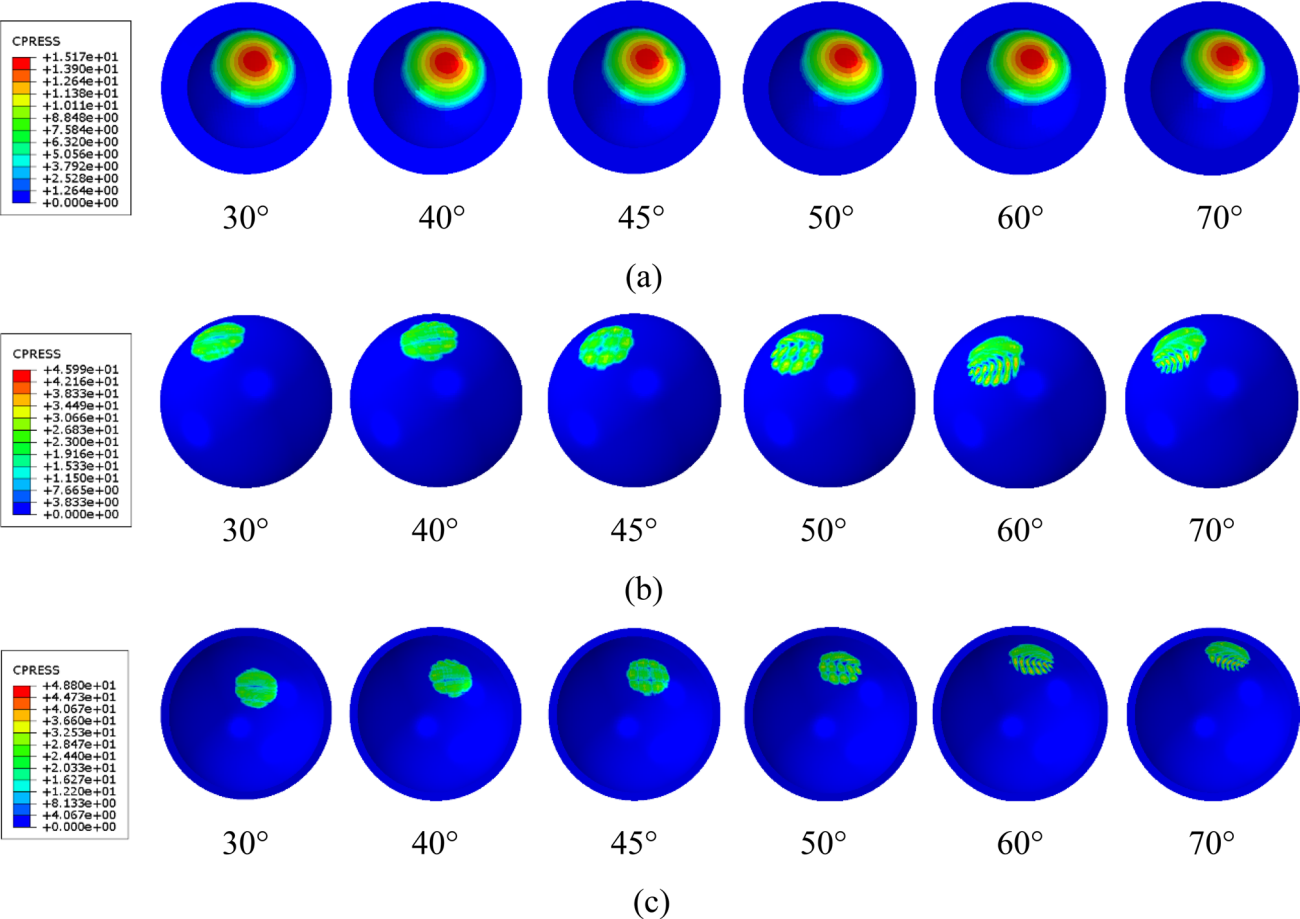
Contour plot of contact pressure on current dual-mobility hip joint prosthesis components: (**a**) inner surface of inner liner, (**b**) outer surface of outer liner, (**c**) inner surface of acetabular cup.

The contour distributions of the maximum contact pressure on the inner surface of the inner liner, the outer surface of the outer liner, and the inner surface of the acetabular cup are shown in Fig. 5a–c, respectively, during the walking gait cycle under several variations in the inclination angle applied on the acetabular cup. The inner and outer liners have the same position, while the acetabular cup has different inclination angles. All the data shown were obtained when the gait cycle reached approximately 65% based on Paul’s gait cycle^35^ since peak loading of the gait cycle occurs at this moment. This means that the highest contact pressure occurs during the peak loading of the gait cycle.

**Figure 6:**
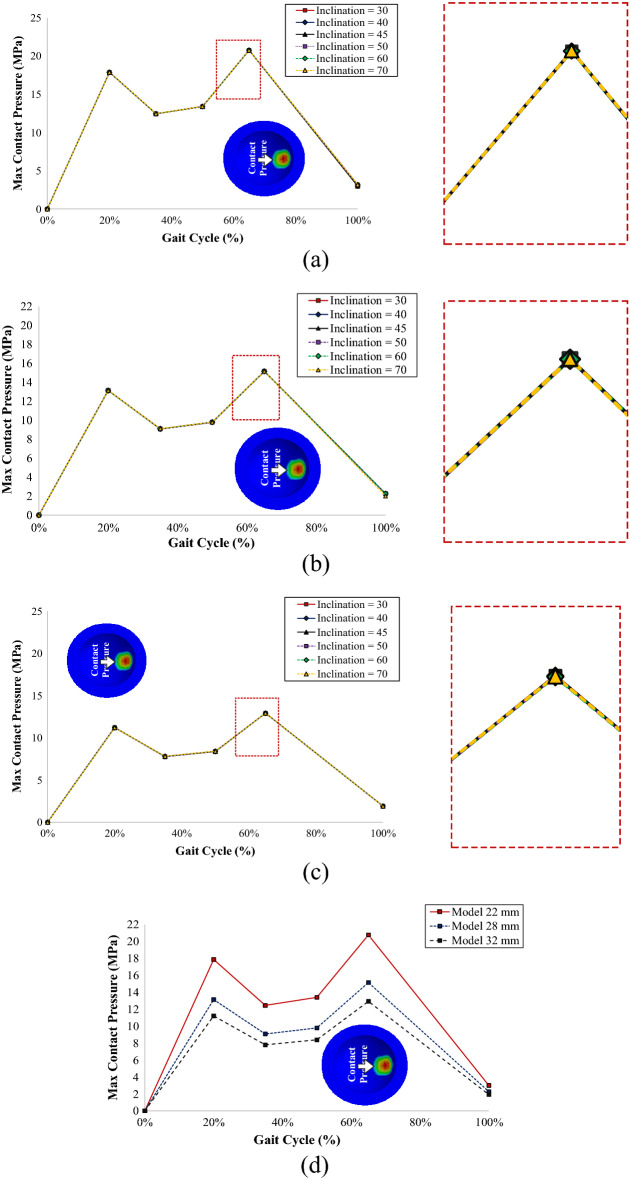
Contact pressure on the inner surface of the inner liner: (**a**) model with a 22 mm femoral head, (**b**) model with a 28 mm femoral head, (**c**) model with a 32 mm femoral head, (**d**) contact pressure comparison of different head diameters at 45° inclination models.

The maximum contact pressure generated from the inner liner is plotted in Fig. 6a–d, which shows that the inclination applied to the acetabular cup did not affect the maximum contact pressure on the inner surface of the inner liner. Each curve represents one variation of inclination angles applied on an acetabular cup. There were slight differences in the inner surface of the inner liner maximum contact pressure of each inclination angle applied on the acetabular cup. It can be seen in Fig. 6a–c that all the curves show coinciding results.

Figure 6a–d depicts the maximum contact pressure on the inner surface of the inner liner for each variation in femoral head diameters of 22 mm, 28 mm, and 32 mm, respectively. The maximum contact pressures on the 22 mm, 28 mm, and 32 mm femoral head diameter models with 65% progress of the gait cycle are 20.78 MPa, 15.16 MPa, and 12.94 MPa, respectively. The 22 mm model generates the highest contact pressure between all diameter variations, followed by the 28 mm and 32 mm models. The maximum contact pressure of every model reaches the maximum point at 65% progress of the gait cycle because of the three-dimensional concentrated forces that maxed out at that condition based on Paul^35^.

**Figure 7:**
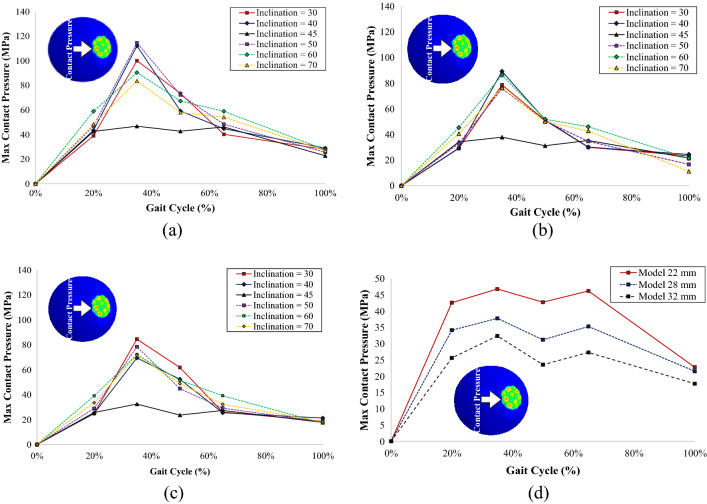
Contact pressure on the outer surface of the outer liner: (**a**) model with a 22 mm femoral head, (**b**) model with a 28 mm femoral head, (**c**) model with a 32 mm femoral head, (**d**) contact pressure comparison of different head diameters at 45° inclination models.

**Figure 8:**
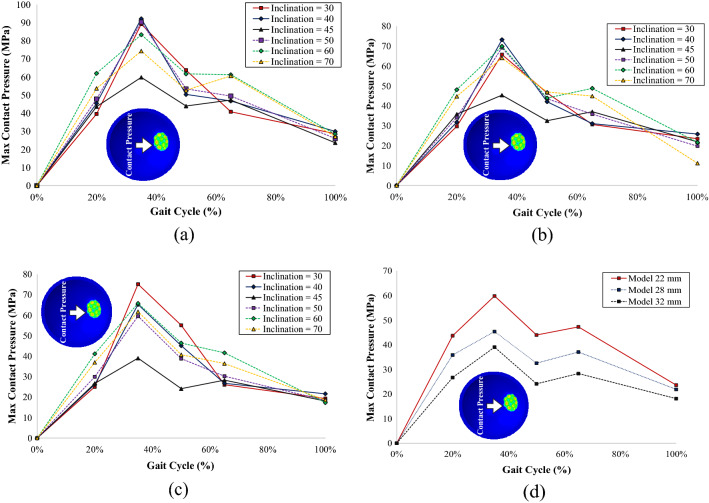
Contact pressure on the inner surface of the acetabular cup: (**a**) model with a 22 mm femoral head, (**b**) model with a 28 mm femoral head, (**c**) model with a 32 mm femoral head, (**d**) contact pressure comparison of different head diameters at the 45° inclination model.

Figures 7 and 8 show that cup inclination affected the maximum contact pressure generated on both the outer surface of the outer liner and the inner surface of the acetabular cup. The 45° inclination model curve on both components has the least fluctuation when compared with other curves with different inclination angles applied. The 45° inclination caused all three components (inner liner, outer liner, and acetabular cup) to be uniformly inclined. Three identical positions of components created a larger contact area that affected the maximum contact pressures generated during the simulation. Korduba et al.^38^ stated that a larger contact area affected by the inclination position (abduction) of the cup will result in a smaller generated contact pressure. These data showed similar agreement in all types of models with different femoral head diameters.

The size variations in all components of the dual-mobility hip joint prosthesis affected the value of the generated contact pressure of each component. A larger femoral head resulted in generally lower contact pressure on all components. All the comparisons of contact pressure generated by all three components on each size variation can be seen in Figs. 6, 7, and 8. Based on the data shown in Figs. 6, 7, and 8, the larger size of the femoral head generally led to generating lower contact pressure on the three components (inner liner, outer liner, and acetabular cup).

## Discussion

The dual-mobility hip joint prosthesis model was used to reduce wear of the implant compared to a single-mobility hip joint prosthesis that has been proven in both mechanical^39^ and medical^40^ perspectives. According to Jamari et al.^29^, contact pressure has a linear correlation with wear on implant bearings; therefore, contact pressure studies can indicate the intensity of wear on implant bearings. The maximum contact pressure generated from the inner surface of the inner liner, the outer surface of the outer liner, and the inner surface of the acetabular cup at a 45° inclination showed a lower contact pressure value than the other inclination models (30°, 40°, 50°, 60°, and 70°) for the same femoral head diameter, whether 22 mm, 28 mm, or 32 mm. Maximum contact pressure graphs generated from the inner surface of the inner liner for each femoral head diameter were not affected by the acetabular cup inclinations. The curves on the graph that represent each inclination angle tend to coincide with each other. These results proved that the inclination angle variations did not have a major effect on the contact pressure. The maximum contact pressure graphs from the outer surface of the outer liner showed that the cup inclinations were sufficient to affect the resulting contact pressure. The curve when the inclination angle of the model was 45° showed the least fluctuating results. The difference between the highest and the lowest contact pressure under the gait cycle is not too large, where it is not more than 10 MPa compared with other models with each inclination angle applied. This means that the 45° inclination can minimize the edge contact that causes strip wear^41^. The contact pressure value at the 45° inclination has the lowest contact pressure under the gait cycle compared to other inclination angles in this study, which indicates that it can minimize wear and extend the life of the implant^22,29^. The resulting contact pressure on the cup's inner surface showed the same curve trend as the curves on the outer liner data. Variations in inclination angle caused a major effect on cup contact pressure when the model was subjected to load. All models except the 45° inclination models showed more fluctuations on the data graphs.

In this modeling, variations in inclination angle only caused a small effect on the resulting contact pressure. The inner liner component in the current model had a more stable contact pressure magnitude under the gait cycle than the liner (without the outer liner) from the conventional dual-mobility hip implant model by Gao et al.^31^. The existence of an outer liner that covered the outer surface of the inner liner based on the implant design according to Saputra et al.^16^ affected the overall resulting contact pressure value of all components, especially the inner liner. It can be seen from the data graphs that all the curves representing the model with one variation in inclination angle coincide with each other so that all the curves look like a single line.

The maximum contact pressure value on the acetabular cup compared to the model by Gao et al.^31^ was generally higher. The presence of the outer liner component caused a lower maximum contact pressure value on the inner liner in the present dual-mobility hip joint prosthesis model compared to the liner (without outer liner) from Gao et al.^31^. Models with an inclination angle of 45° for each femoral head diameter tended to have lower contact pressure on the outer liner and acetabular cup than models with other inclinations. This could happen because when the cup inclination was 45°, all three components of the implant model (inner liner, outer liner, and acetabular cup) would have the same position and inclination. The three components will cover each other’s surface so that the contact area on each component's surface will be more “connected”.

The contact pressure of the present dual-mobility hip joint prosthesis was also compared with a previous study by Uddin^42^, which used a 22.2 mm femoral head design. With variations in the inclination angle of the acetabular cup component of 45°, 50°, 55°, and 60°, the maximum contact pressures on the inner surface of the liner (not using the outer liner) from Uddin^42^ were 21.57 MPa, 21.27 MPa, 21.76 MPa, and 22.19 MPa, respectively. This result is greater than the present dual-mobility hip joint prosthesis design for all geometric variations (22 mm, 28 mm, and 32 mm) with the same acetabular cup inclination. This indicates that the current design demonstrates improved performance in terms of minimizing failures due to wear. In addition, because the current design uses an outer liner to cover the polyethylene liner (as an inner liner), it can reduce the possibility of negative body reactions caused by polyethylene wear particles. The outer liner in the present study is also a design advantage that is not available in previous dual-mobility hip joint prosthesis designs, such as those established by Uddin^42^ and Gao et al.^31^.

The degree of inclination applied to the acetabular cup will affect the contact area. Based on the study by Korduba et al.^38^, an abduction angle of 0° applied to the component resulted in the lowest contact pressure value on the component. An abduction (inclination) of 0° caused the contact surface area to improve so that the contact pressure generated would be smaller. This also follows the data obtained in the current study; when a 45° inclination is applied to the cup, it will generally cause a lower maximum contact pressure for all components (inner liner, outer liner, and acetabular cup). The results from the current study show good agreement with Korduba et al.^38^; when 0° of abduction was applied to the component, it would result in lower contact pressure due to a larger contact area. A larger contact area would cause more “connected” contact for each component of the implant model.

There are several limitations in the present dual-mobility hip joint prosthesis that can affect the contact pressure values obtained. First, the current model only considers bearing components consisting of the acetabular cup, outer liner, inner liner, and femoral head. However, the current model does not consider the influence of the pelvic bone and the role of acetabular fixation, making the computational simulation results less accurate because they are simplified to pursue faster computational simulation completion times^43,44^. Second, in actual conditions, hip joint implants are lubricated with synovial fluid, which is a natural human joint lubricant. Unfortunately, the current model still maintains dry contact by negating the influence of the synovial fluid^45,46^. Next, the materials used—SS 316L and UHMWPE—are assumed to be linearly elastic. UHMWPE should be assumed to be nonlinearly plastic considering its plastic strain properties^47,48^. Moreover, the present computational contact pressure prediction does not conduct sensitivity studies. It is an important step to evaluate potential inaccuracies in the estimate of model inputs (such as material properties) influence model predictions and the conclusions made from the analysis of these predictions. Furthermore, the gait cycle in the present study was adopted from Paul^35^ with quasistatic force at specific time points to simplify the computational configuration. Dynamic force at the complete cycle is required to obtain more realistically accurate contact pressure results on dual-mobility hip joint prostheses^49,50^. Finally, the present results were only validated by Gao et al.^31,37^ on computational simulation results on single- and dual-mobility hip joint prostheses, but a comparison with corresponding experiments was not performed in the present study. Future research on dual-mobility hip joint prostheses is urgently needed to complement the literature from the current lack of research.

## Conclusions

The variations in inclination angle did not affect the inner liner's maximum contact pressure during the gait cycle. There were slight differences in the inner liner maximum contact pressure of each inclination angle applied on the acetabular cup that caused nearly coinciding plotted data charts. On the other hand, the outer liner and cup were affected by the inclination angle applied, creating less fluctuation on the 45° inclination model curve on every variation of femoral head diameter. It can be concluded that the maximum contact pressure of the inner surface of the outer liner on the current FE model shows more stable results compared to the conventional dual-mobility hip joint prosthesis. The least fluctuating outer liner and cup contact pressure curves on the 45° inclination models were caused by the same position of all three components. Such a condition creates a larger contact area for each interacting component, which affects the maximum contact pressure. The present model of a dual-mobility hip joint prosthesis using a 32 mm diameter femoral head with a 45° acetabular cup inclination angle has a better ability to reduce wear.

## Data Availability

All data generated or analyzed during this study are included in this published article.
